# Novel tretinoin 0.05% lotion for the once‐daily treatment of moderate‐to‐severe acne vulgaris in a preadolescent population

**DOI:** 10.1111/pde.13744

**Published:** 2019-01-18

**Authors:** Lawrence F. Eichenfield, Jeffrey L. Sugarman, Eric Guenin, Susan Harris, Varsha Bhatt

**Affiliations:** ^1^ Departments of Dermatology and Pediatrics University of California, San Diego School of Medicine and Rady Children's Hospital San Diego California; ^2^ University of California San Francisco California; ^3^ Ortho Dermatologics Bridgewater New Jersey; ^4^ Bausch Health Bridgewater New Jersey; ^5^ Dow Pharmaceutical Sciences Inc. Petaluma California

**Keywords:** acne vulgaris, preadolescents, topical, tretinoin

## Abstract

**Background:**

Acne vulgaris (acne) is a common skin condition in children and adolescents. Efficacy of tretinoin is well documented in studies that included pediatric patients (12‐18 years of age). With acne routinely presenting in younger patients, data are needed in this important group. Lotion formulations are commonly used across dermatology and are well liked by patients.

**Objective:**

To evaluate the safety and efficacy of a novel once‐daily tretinoin 0.05% lotion in preadolescent subjects (≤ 13 years) with moderate‐to‐severe acne.

**Methods:**

Post hoc analysis of two multicenter, randomized, double‐blind, vehicle‐controlled phase 3 studies in moderate‐to‐severe acne. Preadolescent subjects (N = 154) randomized (1:1) to receive tretinoin 0.05% lotion or vehicle, once daily for 12 weeks. Efficacy assessments included changes in baseline inflammatory/noninflammatory lesions and treatment success (at least 2‐grade reduction in Evaluator's Global Severity Score [EGSS] and clear/almost clear). Safety, adverse events (AEs), and cutaneous tolerability evaluated throughout.

**Results:**

At Week 12, mean percent reduction in inflammatory and noninflammatory lesion counts were 49.5% and 44.0% compared with 31.4% and 18.8% with vehicle (both *P* = 0.001). Treatment success was achieved by 23.7% of subjects by Week 12, compared with 7.2% (*P* = 0.009). The majority of AEs were mild and transient: most frequently were application site pain (5.6%) and application site dryness (2.8%). Local cutaneous safety and tolerability assessments were generally mild‐to‐moderate and improved by Week 12.

**Conclusions:**

Tretinoin 0.05% lotion was significantly more effective than vehicle in achieving treatment success and reducing inflammatory and noninflammatory lesions in preadolescent acne. It was well tolerated, with all treatment‐related AEs deemed mild or moderate.

## 1 INTRODUCTION

Preadolescent acne (≤ 13 years) is common and may be one of the first signs of pubertal maturation. The trend to onset of adrenarche and menarche at an earlier age mirrors the increasingly younger age of acne onset. Comedones tend to predominate, but inflammatory lesions can also be present. Preadolescent acne severity may also be predictive of future disease severity, especially if it remains untreated until the patient is older.^1^ Treatment of preadolescent acne is therefore important not only to ameliorate the development of more severe acne but also to reduce physical and emotional impact and perhaps improve adherence in later life.

Data related to treatment of preadolescent acne are limited, with most medications approved for age 12 and above; off‐label prescribing is common. Topical retinoids (eg, tretinoin, adapalene) have played an essential role in acne management because they inhibit the development of microcomedones and new acne papules.^2^, ^3^ However, there is a common perception that they are primarily effective in comedonal acne,^4^ and their use is associated with significant cutaneous irritation.^5^, ^6^

A new lotion formulation of tretinoin 0.05% has recently been developed leveraging polymerized emulsion technology with the aim to improve both efficacy and tolerability of tretinoin. Here, a post hoc analysis of two large phase 3 clinical studies (N = 1640) evaluates its safety and efficacy in a preadolescent acne population with moderate‐to‐severe acne.

## 2 METHODS

### 2.1 Study design

This was a post hoc analysis of two identical multicenter, randomized, double‐blind, vehicle‐controlled, parallel group clinical studies in pediatric subjects with moderate‐to‐severe acne. Protocols received approval before patient enrollment from the appropriate institutional review board (IRB) and were conducted in accordance with the Declaration of Helsinki and Good Clinical Practices (GCP) and in compliance with local regulatory requirements. All subjects were informed of the study details and provided written consent.

### 2.2 Study population

Eligible subjects included male and female patients of any race and ethnicity aged 9‐13 years who presented with 20‐40 inflammatory lesions (papules, pustules, and nodules), 20‐100 noninflammatory lesions (open and closed comedones), and two nodules or less. A washout period of 2‐4 weeks was required for subjects who used previous prescription and over‐the‐counter acne treatments.

Subjects were enrolled with an Evaluator Global Severity Score (EGSS) of 3 (moderate) or 4 (severe); randomized (1:1) to tretinoin 0.05% lotion or matched vehicle lotion applied once daily for 12 weeks. They were instructed to gently wash their faces with approved cleanser and warm water, rinsing thoroughly and gently patting their face dry. During the study, each subject was permitted to use only approved nonmedicated cleansers, moisturizers, and sunscreens. Lists of approved cleanser and moisturizers were provided. Subjects applied a pea‐sized amount of study drug, dotted on six facial areas, and gently rubbed into the skin covering the entire face (excluding mouth, eyes, inside nose, and lips). Study drug was applied the same time of day, once daily for 12 weeks.

### 2.3 Efficacy evaluation

Efficacy evaluations comprised inflammatory and noninflammatory lesion counts and an EGSS assessment at screening, baseline, and subsequent study visits (Weeks 4, 8, and 12). Efficacy end points included mean percent change from baseline to Week 12 in inflammatory and noninflammatory lesion counts and the proportion of subjects achieving at least a 2‐grade reduction from baseline EGSS and “clear” or “almost clear” at that same visit.

Additional assessments included a patient satisfaction score (PSS) and a validated acne‐specific quality of life (Acne‐QoL) questionnaire (Merck & Co., Inc. Whitehouse, NJ). At baseline, subjects were asked to rate their satisfaction with prior acne therapy with a PSS of 1‐10 (where 10 was the most satisfied and a score of 5 or greater was considered as “satisfied”). At Week 12, they were asked to rate their level of satisfaction with study treatment. Subjects assessed facial shine/oiliness at baseline and Week 12 using a 4‐point scale, and those recording a score of 1‐3 were asked to rate degree of bothersomeness (using a 5‐point scale).

### 2.4 Safety evaluation

Cutaneous safety (erythema and scaling) and tolerability (itching, burning, and stinging) were evaluated on a scale from 0 (none) to 3 (severe). Adverse events (AEs) were evaluated throughout; severity and relationship with study medication were assessed.

### 2.5 Statistical analysis

The intent‐to‐treat (ITT) population comprised all subjects randomized and provided with study drug and vehicle. The safety population comprised all randomized subjects who were presumed to have used the study medication or vehicle at least once and who provided at least one postbaseline evaluation. The primary method of handling missing efficacy data in the ITT analysis set was based on estimation using the Markov chain Monte Carlo multiple imputation methods. No imputations were made for missing safety data.

Treatment comparisons of percent reductions in lesions counts utilized a ranked analysis of covariance with factor of treatment and the respective baseline lesion count as covariate. Significance of EGSS reductions was obtained from logistic regression (using Firth's Penalized Likelihood) with factors of treatment group. There was no imputation of missing data for quality of life analyses. Mean Acne‐QoL scores at Week 12 were compared using an analysis of covariance with factors of treatment and the respective baseline lesion count as covariate. Shininess/oiliness and PSS scores were compared between treatment groups using a Wilcoxon Rank Sum test. All statistical analyses were conducted using SAS^®^ version 9.3 or later. Statistical significance was based on 2‐tailed tests of the null hypothesis resulting in *P* values of 0.05 or less.

All AEs occurring during the studies were recorded and classified on the basis of medical dictionary for drug regulatory activities terminology (MedDRA) for the safety population. Treatment emergent adverse events (TEAEs), defined as any AE with an onset on or after the date of first drug application, were summarized by treatment group and relationship with study drug. Each subject was counted only once within a system organ class or a preferred term using the event with the greatest severity or causality, respectively.

## 3 RESULTS

### 3.1 Baseline characteristics

**Figure 1:**
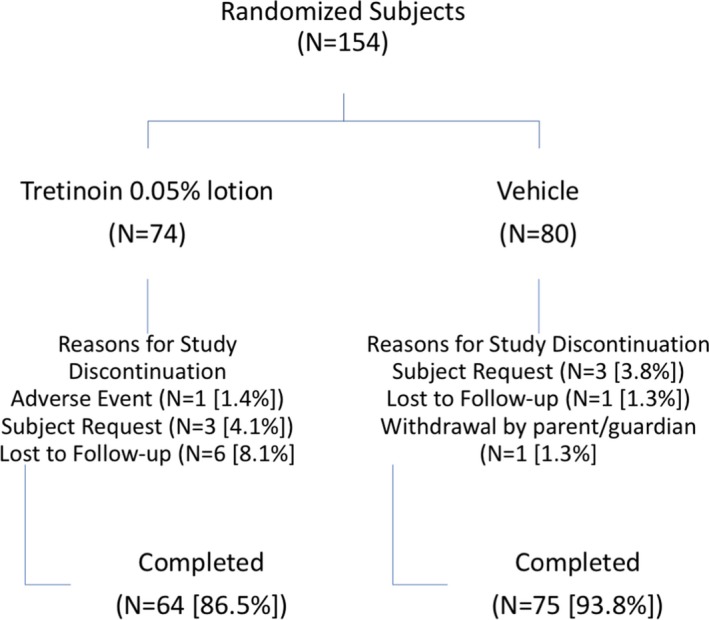
Patient disposition showing percent completion and reasons for discontinuation (ITT pediatric population pooled data)

One hundred fifty‐four subjects were included in the post hoc analysis. Of those, 139 (90.3%) completed the studies, including 64 preadolescent subjects (86.5%) on tretinoin 0.05% lotion and 75 (93.8%) on vehicle (Figure 1). The most common reasons for study discontinuation were “lost to follow‐up” or “subject request.”

Demographic data (Table 1) were similar across the treatment groups. Mean age (standard deviation [SD]) was 12.4 (0.94) years. Subjects were predominantly White (74.7%) and women (59.1%).

**Table 1:** Demographics and baseline characteristics (ITT pediatric population, pooled data)

	Study 301 + 302 (pooled data pediatric population)		
	Tretinoin 0.05%, (N = 74)	Vehicle, (N = 80)	Total, (N = 154)
Age—Mean years (SD)	12.4 (0.97)	12.4 (0.92)	12.4 (0.94)
Range	9‐13	9‐13	9‐13
Sex N (%)			
Male	31 (41.9%)	32 (40.0%)	63 (40.9%)
Female	43 (58.1%)	48 (60.0%)	91 (59.1%)
Ethnicity N (%)			
Hispanic or latino	17 (23.0%)	24 (30.0%)	41 (26.6%)
Not hispanic or latino	57 (77.0%)	56 (70.0%)	113 (73.4%)
Race N (%)			
American Indian or Alaska Native	0 (0.0%)	0 (0.0%)	0 (0.0%)
Asian	3 (4.1%)	2 (2.5%)	5 (3.2%)
Black or African‐American	14 (18.9%)	15 (18.8%)	29 (18.8%)
Native Hawaiian or Other Pacific Islander	0 (0.0%)	2 (2.5%)	2 (1.3%)
White	55 (74.3%)	60 (75.0%)	115 (74.7%)
Other	2 (2.7%)	1 (1.3%)	3 (1.9%)
Evaluator's global severity score N (%)			
3—Moderate	72 (97.3%)	73 (91.3%)	145 (94.2%)
4—Severe	2 (2.7%)	7 (8.8%)	9 (5.8%)
inflammatory lesion count—Mean (SD)	26.2 (5.50)	27.2 (6.49)	26.7 (6.04)
Noninflammatory lesion count—Mean (SD)	48.3 (22.01)	52.1 (21.78)	50.3 (21.90)

There were no noticeable differences between treatment groups with regard to lesion counts or EGSS. At baseline, the mean number (SD) of inflammatory and noninflammatory lesions was 26.7 (6.04) and 50.3 (21.90); 94.2% of subjects had moderate acne (EGSS = 3).

### 3.2 Efficacy

#### 3.2.1 Lesion counts

**Figure 2:**
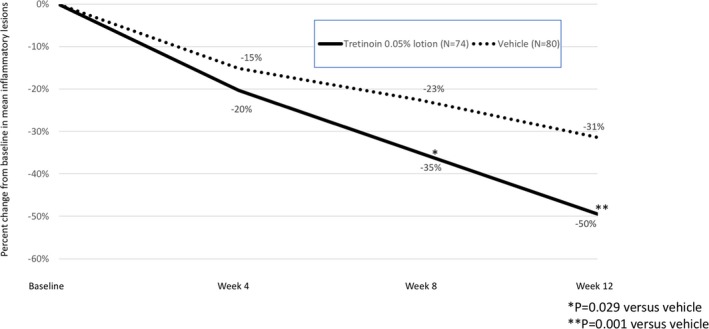
Percent change in inflammatory lesions from baseline to Week 12 (ITT pediatric population pooled data, LS Mean)

**Figure 3:**
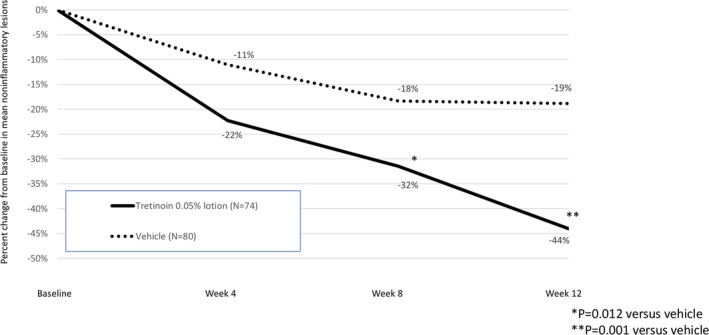
Percent change in noninflammatory lesions from baseline to Week 12 (ITT population pooled data, LS Mean)

Tretinoin 0.05% lotion resulted in statistically significant reductions in inflammatory and noninflammatory lesion counts compared to vehicle from Week 8 through Week 12. Notably, there was no additional improvement seen with vehicle beyond the reduction in noninflammatory lesion counts reported at Week 8. Mean percentage change (LS mean) from baseline to Week 12 in inflammatory and noninflammatory lesion counts was 49.5% and 44.0% compared with 31.4% and 18.8% with vehicle (both *P* = 0.001); see Figures 2 and 3.

#### 3.2.2 Treatment success

**Figure 4:**
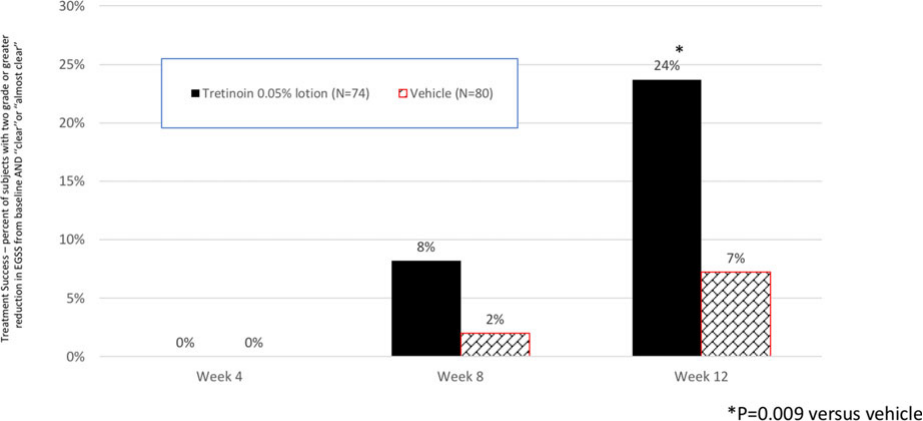
Treatment success. Subjects with at least a 2‐grade improvement and “clear” or “almost clear” at each study visit (ITT population pooled data)

By Week 12, 23.7% of subjects were treatment successes (at least a 2‐grade improvement in global severity by EGSS and “clear” or “almost clear”) following treatment with tretinoin 0.05% lotion compared to only 7.2% on vehicle (*P* = 0.009); see Figure 4.

#### 3.2.3 Patient satisfaction and quality of life

Subject satisfaction with treatment was significantly greater with tretinoin 0.05% lotion than vehicle by Week 12 (*P* = 0.008). Although there were no statistically significant differences in the improvement between treatment groups based on the mean Acne‐QoL assessments in each of the four evaluated domains, the increases in mean scores from baseline to Week 12 with tretinoin 0.05% lotion were greater than vehicle for self‐perception (3.7 vs 3.5), role‐emotional (3.4 vs 3.0), and acne symptoms (3.7 vs 3.0).

### 3.3 Safety

A similar number of subjects in each treatment group (22 and 18, tretinoin 0.05% lotion and vehicle, respectively) reported treatment emergent (TE) AEs. All TEAEs experienced in the tretinoin 0.05% lotion group were mild or moderate events (Table 2).

**Table 2:** Safety: Treatment emergent and related adverse event (AE) characteristics through Week 12 (safety pediatric population, pooled data)

	Tretinoin 0.05% lotion	Vehicle lotion
Subjects reporting any TEAE	22 (30.6%)	18 (23.1%)
Subjects reporting any SAE	0 (0.0%)	0 (0.0%)
Subjects who died	0 (0.0%)	0 (0.0%)
Subjects who discontinued due to TEAE	1 (1.4%)	0 (0.0%)
Severity of AEs reported		
Mild	11 (15.3%)	11 (14.1%)
Moderate	11 (15.3%)	4 (5.1%)
Severe	0 (0.0%)	3 (3.8%)
Relationship with study drug		
Related	7 (9.7%)	1 (1.3%)
Unrelated	15 (20.8%)	17 (21.8%)
Treatment‐related AEs reported by ≥ 1% subjects		
Application site pain	4 (5.6%)	0 (0.0%)
Application site dryness	2 (2.8%)	0 (0.0%)
Application site exfoliation	1 (1.4%)	1 (1.3%)
Application site pruritus	1 (1.4%)	0 (0.0%)
Application site irritation	1 (1.4%)	0 (0.0%)

Treatment‐related AEs reported by ≥ 1% of subjects treated with tretinoin 0.05% lotion were application site pain (5.6%); dryness (2.8%); and irritation, exfoliation, or pruritus (each 1.4%).

### 3.4 Cutaneous safety and tolerability

**Figure 5:**
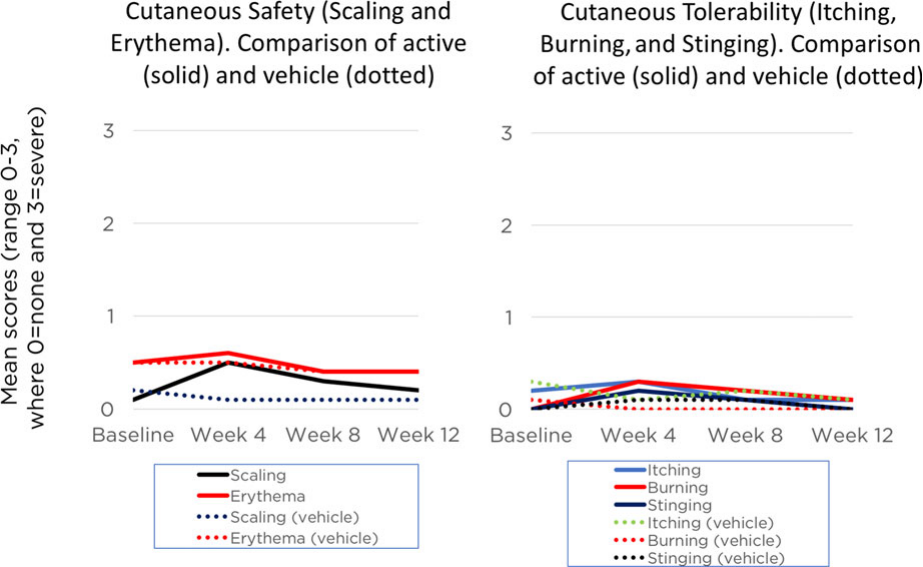
Cutaneous safety and tolerability assessment from baseline to Week 12 (Safety pediatric population, pooled data)

Erythema and scaling were recorded by the investigator. Overall mild‐to‐moderate erythema was noted in 43% of subjects at baseline, with 12% reporting mild‐to‐moderate scaling. Mean scores for both erythema and scaling increased at Week 4 (to 0.6 and 0.5, respectively, where 0 = mild) following treatment with tretinoin 0.05% lotion, returning to baseline levels by the end of the study (Figure 5).

Levels of itching, burning, and stinging were obtained from the subjects. Overall, 19% of subjects reported mild‐to‐moderate itching at baseline. Only four subjects (6%) reported any itching by Week 12. Reports of burning (N = 7, 5%) and stinging (N = 6, 4%) were infrequent and mild at baseline. Mean scores increased very slightly at Week 4 (to 0.3 and 0.2, respectively, where 0 = mild) with tretinoin 0.05% lotion, again returning to baseline levels by Week 12.

## 4 DISCUSSION

Topical retinoids are routinely used to treat acne; safety and efficacy are well documented in large pivotal trials. With limited FDA‐approved treatment options for preadolescent acne, off‐label prescribing is common. Monotherapy with adapalene gel, tretinoin gel, and tretinoin microsphere gel has been investigated in small open‐label and blinded studies in children under 12 years of age.^7^, ^8^, ^9^ However, data in preadolescent acne are lacking.

In a 12‐week open‐label study of 40 children (8‐12 years) with mild‐to‐moderate acne, tretinoin microsphere 0.04% gel produced a significant decrease in EGSS from baseline to Week 12 (from 2.6 to 2.1, *P* < 0.0001). Eight patients (22.2%) were “clear” or “almost clear.” Skin irritation occurred in 35% of patients, being generally mild and transient.^8^ In a subsequent double‐blind study with tretinoin microsphere 0.04% gel in 110 children (9‐11 years) with moderate acne, there was a significantly greater improvement in noninflammatory lesions (44.0% reduction) compared to vehicle (30.8%, *P* = 0.04) at Week 12.^9^ In a post hoc analysis of two studies comparing tretinoin microsphere 0.1% gel and tretinoin 0.05% gel in young adolescents (10‐14 years) with mild‐to‐moderate acne, comparable lesion reduction and treatment success were noted. Tretinoin 0.05% gel demonstrated better cutaneous tolerability.^10^ Fourteen percent of participants reported dry skin, 8% skin burning sensation, 5% erythema, and 5% dermatitis exfoliative compared with 32%, 11%, 23%, and 23%, respectively, with tretinoin gel microsphere 0.1% (all *P* < 0.001, except skin burning sensation).

Tretinoin 0.05% lotion is a novel topical treatment for moderate‐to‐severe acne leveraging polymerized emulsion technology. The development rationale was to provide a tretinoin formulation with improved efficacy and tolerability that could be especially suited to a preadolescent population. The polymerized emulsion provides a mesh (a polymeric network) which helps structure the emulsion providing uniform distribution of active and hydrating ingredients at the surface of the skin, reducing the presence of concentrated drug in specific areas (hot spots). It also forms a barrier which helps keep the skin hydrated by reducing epidermal water loss and increasing skin water content.

Tretinoin 0.05% lotion was shown to provide significantly greater efficacy than vehicle in two pivotal phase 3 studies.^11^ Data reported herein were consistent with these overall findings. Tretinoin 0.05% lotion offered significantly more efficacy than vehicle in inflammatory and noninflammatory lesion reduction and treatment success, with greater patient satisfaction (compared with vehicle) and preference (compared with previous acne therapy). Efficacy in comedonal acne (44% mean reduction) was similar to that reported previously with tretinoin microsphere 0.04% gel,^9^ although our analysis included preadolescent subjects with severe disease. To our knowledge, we are the first to report efficacy in inflammatory lesions (50% mean reduction). Results in this pediatric population were similar to those seen in the overall study populations. The only difference of note is that the vehicle tended to be less effective in the pediatric population.

In the overall study populations, tretinoin 0.05% lotion showed significantly greater QoL benefits relative to vehicle. Although there were numerical differences in our post hoc analysis, they were not significant and may reflect the appropriateness of some of the individual questions to a preadolescent acne population, given the relatively low mean scores are baseline compared with the overall study populations.

Tretinoin 0.05% lotion was generally safe and very well tolerated.^11^ The most commonly reported treatment‐related AEs included application site reactions and skin‐related events attributed to the known properties of tretinoin. There were slightly more treatment‐related AEs when compared to the overall study populations.

Most noteworthy was the extremely low irritation potential of this novel tretinoin formulation. The level of irritation seen with tretinoin 0.05% lotion in our study appears lower than that reported in clinical studies with tretinoin microsphere gel (0.04% or 0.1% concentrations) or tretinoin gel 0.05% in preadolescent acne;^8^, ^9^, ^10^ however, direct comparisons are difficult to make in the absence of head‐to‐head trials.

## 5 CONCLUSION

A novel tretinoin 0.05% lotion formulation was an effective and well‐tolerated topical treatment for moderate‐to‐severe comedonal and inflammatory preadolescent acne.

## CONFLICT OF INTEREST

LFE and JLS were study investigators and are advisors for Ortho Dermatologics. EG, SH, and VB are employees of Bausch Health.
